# Ethical and legal implications of whole genome and whole exome sequencing in African populations

**DOI:** 10.1186/1472-6939-14-21

**Published:** 2013-05-28

**Authors:** Galen EB Wright, Pieter GJ Koornhof, Adebowale A Adeyemo, Nicki Tiffin

**Affiliations:** 1 South African National Bioinformatics Institute, University of the Western Cape, Bellville, South Africa; 2 Department of Mercantile and Labour Law, University of the Western Cape, Bellville, South Africa; 3 Center for Research on Genomics and Global Health, National Institutes of Health/National Human Genome Research Institute, Bethesda, MD, USA

**Keywords:** African populations, Ethical, legal, and societal issues, Next generation sequencing, Whole genome and whole exome sequencing

## Abstract

**Background:**

Rapid advances in high throughput genomic technologies and next generation sequencing are making medical genomic research more readily accessible and affordable, including the sequencing of patient and control whole genomes and exomes in order to elucidate genetic factors underlying disease. Over the next five years, the Human Heredity and Health in Africa (H3Africa) Initiative, funded by the Wellcome Trust (United Kingdom) and the National Institutes of Health (United States of America), will contribute greatly towards sequencing of numerous African samples for biomedical research.

**Discussion:**

Funding agencies and journals often require submission of genomic data from research participants to databases that allow open or controlled data access for all investigators. Access to such genotype-phenotype and pedigree data, however, needs careful control in order to prevent identification of individuals or families. This is particularly the case in Africa, where many researchers and their patients are inexperienced in the ethical issues accompanying whole genome and exome research; and where an historical unidirectional flow of samples and data out of Africa has created a sense of exploitation and distrust. In the current study, we analysed the implications of the anticipated surge of next generation sequencing data in Africa and the subsequent data sharing concepts on the protection of privacy of research subjects. We performed a retrospective analysis of the informed consent process for the continent and the rest-of-the-world and examined relevant legislation, both current and proposed. We investigated the following issues: (i) informed consent, including guidelines for performing culturally-sensitive next generation sequencing research in Africa and availability of suitable informed consent documents; (ii) data security and subject privacy whilst practicing data sharing; (iii) conveying the implications of such concepts to research participants in resource limited settings.

**Summary:**

We conclude that, in order to meet the unique requirements of performing next generation sequencing-related research in African populations, novel approaches to the informed consent process are required. This will help to avoid infringement of privacy of individual subjects as well as to ensure that informed consent adheres to acceptable data protection levels with regard to use and transfer of such information.

## Background

The rapid progress of high throughput genomic technologies and next generation sequencing (NGS) methods in recent years has changed the scope of human genomic studies. These advances have made it feasible to routinely perform whole genome sequencing (WGS) and whole exome sequencing (WES) studies, which together we subsequently refer to using the acronym ‘WGES’. The power of WGES research to generate vast amounts of high-resolution data is unprecedented, and these sequencing methods enable the capture of the full extent of genetic variation in an individual’s genome or their entire gene-coding region respectively. Such approaches have already proven beneficial for the identification of the genetic causes of several Mendelian disorders (for example, see [1-4]); and the adoption of NGS in the research of complex disorders is also becoming more feasible with the corresponding reductions in the cost of these technologies (e.g. National Heart Lung and Blood Institute Exome Sequencing Project) [1]. Emerging WGES studies are therefore extremely diverse in nature and will play an essential role in determining the genetic aspects of human disease susceptibility, risk of complications, modifiers of disease severity, outcomes and response to therapy (including drug response). Further, increasing numbers of researchers are becoming involved in the secondary-use of genomic research. This trend has been fuelled by funding agencies and journals frequently requesting that genomic data and corresponding phenotypic information be shared with interested investigators or made available in controlled access databases in order to ensure that these data are used to their full potential and to expedite the generation of robust results.

Due to the large-scale, collaborative nature of WGES studies, ethical and legal issues are of increasing concern and have important implications for society [2-4]. Since genomic research is global in nature, these issues required nuanced discussions to ensure that procedures and frameworks that are adopted are culturally sensitive and adequate for the settings in which they are conducted. This is especially the case for resource-limited settings, such as those found in Africa, where the implications of such research remain to be fully established. Including sufficient representation of different human populations in genomics research is crucial to understanding the full range of human genomic variation and a means to reducing disparities in genomics research and its applications. African populations are of unique interest because, despite having the highest levels of genetic diversity, living in a vast array of environmental and cultural settings, as well as suffering from a high burden of disease that could be studied using genomics approaches, they have been underrepresented in past research [5,6].

The underrepresentation of Africa in genomic research has led to the launch of programmes, such as the Human Heredity and Health in Africa (H3Africa) Initiative [7], which are funded by the Wellcome Trust (UK) and the National Institutes of Health (NIH, USA) with approximately $37 million of funding over five years. The first round of H3Africa funding will support research into kidney disease, diabetes, heart disease, obesity, tuberculosis and African sleeping sickness; as well as the establishment of biorepositories and a bioinformatics network [8]. Such initiatives should aid in the development of skills and infrastructure required for genomic research and, additionally, due to the decreasing cost of NGS, lead to the sequencing of numerous African genomes and exomes. Simultaneously, these studies raise unique sets of ethical and legal challenges that should be considered in order to ensure the protection of the study participants and their communities, as well as the researchers themselves.

In this study, we analyse ethical and legal considerations for Africa resulting from genomic studies involving WGES, with particular emphasis on informed consent, data sharing, return of research results and secondary/incidental findings, as well as research participant privacy. The right to privacy encompasses the right to control the dissemination of information about one’s private life. It is submitted that the large amount of data made publicly available through WGES studies does pose a potential threat to the privacy of individuals participating in such studies, where true anonymisation cannot always be guaranteed. This is especially exacerbated through disclosure of data in an online environment. This notion, and the extent to which it should be guarded against, shall be expanded upon in this paper. Relevant South African legislation is examined and benchmarked against major foreign jurisdictions such as the USA and the European Union (EU) in order to determine to what extent there is universality, or conversely divergence, on relevant aspects.

This study aims to be a starting point for researchers involved in WGES in African populations by raising some of the ethical and legal considerations that need to be addressed in order to protect the interests of the study participants as well as the researchers. We acknowledge the intricate nature of these concepts and therefore only aim to discuss some of the most pertinent issues raised by these technologies in research settings on the African continent. The issues that need to be addressed before implementing WGES into clinical care in this environment are beyond the scope of this debate.

## Discussion

### Ethical concerns for whole genome and whole exome sequencing studies

#### Informed consent

Genomic studies involving human research subjects typically require ethical clearance from relevant institutional review boards (IRBs) or research ethics committees (RECs) before commencing. This process involves members of the IRBs/RECs determining whether the research protocol will gather sufficient informed consent from study participants to ensure that these individuals are able to make informed decisions regarding potential risks and benefits of voluntary participation [9]. The consent procedure should be an on-going process, which involves more than only an initial informed consent document: researchers should engage prospective study participants in frequent discussions about their research, as well as give them adequate time to decide whether they wish to participate in the proposed research, ensuring that participants realise that participation is voluntary. In certain settings, consultations with the community may also be appropriate (see the section, ‘Challenges and opportunities in the African context’, below).

NIH-funded research involving human subjects is guided by the US Department of Health and Human Services Policy for the Protection of Human Research Subjects Code of Federal Regulations, 45 CFR Part 46 [10]. Of relevance to research in Africa is the fact that the informed consent process of projects funded by the H3Africa Initiative will be required to comply with the so-called ‘Common Rule’ (i.e. subpart A of 45 CFR Part 46) [11]. Published in 1991, a large part of these regulations were derived from concepts from the Belmont Report [12,13], a report that places emphasis on: (i) respect for persons, (ii) beneficence and (iii) justice. The ‘Common Rule’ is the basic policy for the protection of human research subjects for federally funded research in the USA. The policy covers aspects such as general requirements for informed consent and criteria for IRB membership and oversight.

The informed consent document should contain a variety of different elements presented in a concise manner to ensure that participants can easily comprehend the text. Elements normally included are a brief description of the project, the goals of the research, the potential risks and benefits of participation, return of results, options for withdrawal from research and data sharing plans. It is imperative for the informed document to be written in accessible language to promote research participant autonomy and awareness of potential benefits or harms of participation.

#### Data sharing

One of the key aspects of genomic research, which is frequently adopted by these studies (including those studies involving WGES), is the concept of data sharing through databases to allow for the secondary use of data. Such data sharing has been employed in the field of human genomics since the advent of genome wide association studies (GWAS) [14]. Increasing data access has allowed genomic datasets to be thoroughly analysed, and has afforded research groups without data-generating capacity the opportunity to interrogate genomic data.

The related benefits to society of these data sharing practices have led certain public funding agencies as well as journals to require researchers to deposit genomic data and correlated deidentified phenotypic information into public databases. For example, the federally-funded NIH expects research resources generated by NIH projects to be shared with the scientific community unless there are adequate reasons that would justify that exemption (e.g. data sharing not covered in the original consent documents, institutional policies, etc.) [15]. Examples of such databases include the Database of Genotypes and Phenotypes (dbGaP) and the European Genome-phenome Archive (EGA). These databases were originally operated under open access models; this practice, however, is no longer endorsed by the NIH and Wellcome Trust since a study by Homer et al. [16] displayed that certain genotypic information makes it is possible to determine whether a particular individual participated in a GWAS [17]. Currently, these databases are typically operated under restricted/controlled access and data access committees oversee the dissemination of such data.

As illustrated by Homer et al. [16], despite the ‘practical obscurity’ of genomic data, privacy and issues pertaining to identifiability are inherent concerns when genomic data sharing is practiced. Additionally, privacy risks are not exclusive to the genomic research participants themselves, but extend to their families too. Although personal identifiers are removed from genotype-phenotype data in public databases, the risk of the inference of individuals by data intruders still exists due to richness of such data and increasing numbers of different types of public databases [17-21]. Therefore dbGaP and EGA require users requesting to gain access to individual genotype-phenotype information to sign agreements that state that data will not be used to attempt to identify participants and that data confidentiality will be maintained. These are data use certification [22] and data access agreements [23], respectively. It is, however, impossible to guarantee absolute confidentiality to research participants in genomic studies, and it is important that such a caveat is included in the informed consent document. Finally, once the data have been deposited in these databases, withdrawal from research – an option frequently found in informed consent documents – becomes challenging [2].

For these reasons, the sensitive nature of genomic data with regards to identifiability and privacy, as well as stigmatisation and discrimination that could result from data breaches, are major concerns in these studies, while the physical harms of participating in such research are usually minimal (e.g. the pain experienced due to blood draw): unlike traditional clinical research, the major harms of genomic research are not physical, but psychosocial in nature [9]. These risks of data sharing need to be carefully measured against the potential loss of medical advancement to society through withholding the data.

#### Participant identifiability

The deluge of data that are generated in WGES studies differ dramatically from DNA microarray-based GWAS since, theoretically, all the genetic variants that change protein sequences in an individual are catalogued, heightening the ethical and legal concerns encountered in traditional genetic studies. The extensive nature of these WGES data, as well as links to corresponding phenotypic and familial data, increases the likelihood of identifiability of research participants and complicates the informed consent process [24,25]. These concerns provide challenges for IRBs/RECs as well as investigators wanting to conduct WGES studies, since due to the emerging nature of WGES technologies, empirical evidence and policies regarding these concepts are lacking.

Due to these concerns, the contents of the informed consent document come under increased scrutiny in WGES studies. This is of particular relevance to samples that were collected before NGS era of medical genomic research, and IRBs/RECs could conclude that participants need to re-consent to the WGES part of the study. Although the goals of traditional genetic and genomic studies do not differ greatly from WGES studies, there is still some debate whether research participants need to be specifically informed about the use of WGES in a study, and if so, how extensive and in what language the description of the WGES approach should be in the consent document [4,26]. Practicing genomic data sharing is also another potential issue here if the individuals were not advised about this concept in the original informed consent documents [27]. Further, WGES approaches are far more prone to uncover secondary or incidental research findings (i.e. those not related to the primary research aims/scope of the study) and if there is to be an option for such results to be returned to research participants, this should be clarified in the informed consent document [28]. This last concept will be discussed in more detail later.

The use of broad consent has been suggested as a possible solution to allow for genomic data to be shared as well as permitting the inclusion of DNA samples in biorepositories [14,29]. This will come under heightened inspection with the advent of WGES studies, since the use of broad consent models opens concerns about decreased research participant autonomy and may impair their ability to make informed decisions [14,25]. The feasibility of using narrow consent models for biorepositories is questionable; since this would hamper research outputs from these resources and could necessitate excessive re-contact of research participants. Research involving potential biorepository participants in the US indicates that privacy is a notable concern for these individuals and should be addressed in future informed consent documents [30].

The privacy risks associated with WGES data sharing are increased due to the scope of these data, which include information on rare alleles as well as variants of clinical utility that will aid in re-identification of samples. Research has shown that it may be possible to use DNA chip data from the HapMap Project [31] to predict the potential surnames of participants from this initiative [20] and it has recently been displayed that similar strategies, using free, publically available resources, can be employed with personal genome datasets to identify if analysed in conjunction with other available data (e.g. age and place of birth) [21]. Further, as the tools to analyse WGES data improve, profiling of identifiable phenotypic traits will become more straightforward [19,32] and Mendelian diseases, which are frequently included in WGES studies, are also easier to re-identify due to their rarity and unique pedigrees that are often included in publications.

#### Return of research results and secondary/incidental findings

Due to the fact that WGES technologies efficiently analyse the entire genome and exome respectively; secondary, incidental or unrelated findings that were not part of the original research hypothesis may be encountered, which places emphasis on the researchers’ duty to inform participants of such results. The dilemma faced by genetic/genomic researchers in handling such findings is not new, yet the scope of the results generated by WGES exponentially amplifies the probability of encountering these findings exponentially [33]. It has been suggested that ethical obligations and legal duties for returning research results may exist [28,34-38]. For example, Wolf et al. [34] highlight the relevant ethical concepts of reciprocity between researchers and their participants and the welfare and autonomy of the research participants; and legal obligations such as the development of management plans for secondary/incidental results, as well as the implementation of these when required. The moral duty to warn research participants of secondary/incidental variants of interest, however, needs to balanced against opposing duties such as the limits of beneficence, excessive burdens on researchers, and the fact that returning results may be harmful (e.g. cause anxiety) [39].

If an option to return secondary/incidental findings to research participants will be given in a study, it is important to determine what results can justifiably be returned to these individuals in order to minimise risks and increase benefits of such a practice. Since it is not feasible to return all WGES data that have been associated with clinical phenotypes to participants, researchers must determine which type of genetic variants should be returned to research participants. Current thinking suggests that these variants should have clinical relevance, have implications for health and are medically actionable [26,33,40]. Categorical approaches such as the binning of genomic variants according to clinical relevance during interpretation may aid in reducing such data into manageable portions [41]. Expert, confidential consultation sometimes may be required to predict the relevance of particular findings [42] and should be incorporated into the design of the informed consent process [34]. During the informed consent process, it is also important to explain to the participants the implications of returning secondary/incidental findings and then to subsequently determine whether the research participants would opt to receive these results, and what their expectations are in this regard. Further, ensuring analytical validity through additional verification steps and incorporation of genetic counselling are important steps to include in disclosure plans [28].

Despite these recommendations, debate exists with regards to the practice of returning secondary/incidental findings from WGES research and other genomic studies. Some have suggested that the return of research results may distort the line between research and clinical practice [9,37] and that burdensome legal liabilities for researchers may be created [43]. Another complication arises when attempting to determine what results are considered clinically relevant and how substantial the related risks need to be in order to be considered meet the criteria for such return [36,42,44]. This is particularly challenging with novel variants, which is highly relevant for WGES research [4]. In this regard, it is important to draw a distinction between GWAS and WGES studies. GWAS-associated loci for most diseases are often associated with small-to-moderate risk (Odds Ratios of 1.3-1.6) and have low predictive value for an individual. Therefore, their clinical relevance and potential for being actionable are quite limited. WGES studies on the other hand often find coding mutations with large effect sizes, some of which are already annotated as disease-causing (for example in the Human Gene Mutation Database). For instance, it is estimated that healthy individuals carry, on average, approximately >2 robust disease-causing mutations in their genomes [45]. This makes the issue of secondary/incidental findings far more complex in WGES studies.

The aspects discussed above need to be considered when designing qualified results disclosure programs [39] and should be incorporated into future research studies attempting to generate empirical evidence on return of secondary/incidental findings. Initiatives such as My46 [46], which aims to gather participants’ opinions and preferences for the return of WGES research results, should aid in better understanding the intricacies of this concept better. Sets of guidelines and policy statements produced by workgroups from organisations such as the American College of Medical Genetics and Genomics [47] with regards to the application of NGS technologies in clinical settings (including secondary/incidental findings) could also provide useful information for research environments. Additionally, a preliminary study of specialists’ opinions for the management of WGES secondary/incidental findings – ordered for clinical purposes – cited 64 genetic conditions or genes that have strong evidence for return to patients [48]. As such lists emerge, they could help guide research studies. In the interim, it is important for researchers to work intimately with their respective IRBs/RECs during processes such as the review of WGES research protocols.

### Challenges and opportunities in the African context

#### Cultural and genetic diversity in Africa

It is essential to perform WGES studies in Africa to ensure that the benefits of genomic medicine are also eventually realised in these populations. Conducting research in the culturally as well as genetically diverse populations of Africa, however, requires unique considerations to ensure that this research is performed in a manner that is respectful to cultural differences; and community engagement can be helpful in certain situations. Previous large-scale genomics projects involving indigenous African populations, such as the HapMap Project [31], aimed to document unforeseen issues and ensure that culturally sensitive research was achieved through consultation with community advisory groups [49,50]. For example, certain tissues and materials may have unique cultural significance in specific African populations and researchers need to be aware of such facts when collecting samples for genomic research [51,52].

It is important for advisory groups to be representative of the socio-economic makeup of the actual communities [53], yet guidelines for conducting community engagement are notably lacking [54]. The selection of the appropriate members for such advisory groups is therefore a major concern [53-55], and can lead to what has referred to as ‘pseudo-community engagement’ [53]. Despite the fact that appropriate community engagement can be a complex, time-consuming process, it aids in establishing strong collaborations between researchers and research participants [56]. This is in contrast to so-called ‘parachute research’ [57], which does not promote sustainable research conditions and creates feelings of distrust of research amongst the respective communities. If performed correctly, community engagement helps address group concerns, increases the community’s understanding of the respective research, aids in preventing exploitation, enhance recruitment of research participants and helps maintain openness and transparency between researchers and potential study participants. Such processes can also be used to determine how the respective populations would like to be named and described in subsequent research.

Studies are required with regards to opinions of risks associated with identifiability caused by genomic research in the African context. The risks associated with identifiability associated with participating in genomic research will be of increased concern when diseases/disorders that have stigma associated with them in Africa are studied (e.g. mental health [58] and HIV/Aids [59]). Additionally, in Africa individuals frequently define themselves with regards to a community, rather than as a lone individual [60]. Therefore, these differences to Western thought need to acknowledged and the potential for group harm resulting from inclusion of certain ethnic groups needs to considered.

As mentioned above, the results of genetic/genomic research, including WGES studies, may result in concerns about discrimination and stigmatisation due to research findings [61,62]. In the US, Goldenberg et al. [63] found that the potential for genetic research to cause group stigmatisation could influence whether individuals decide to participate in the study, a concern especially apparent amongst African-American respondents. De Vries et al. [64] noted that this genomics-related concern could be relevant for ethnic groups in Africa for which stigmatisation and discrimination is already present. The unfortunately long list of past and on-going ethnic conflict and violence in many African countries suggests that this is a real concern. In other words, genomics findings could be used as one more piece in highlighting perceived differences between ethnic groups that are used to promote such conflicts. On the other hand, it should be noted that other distinguishing characteristics (e.g. in skin colour, height, or lifestyle) are already being so misused. Therefore, it is uncertain if genomics findings would exacerbate such issues than what currently exists or if, in fact, the genomic similarities between ethnic groups will lead to a greater appreciation of the shared humanity of all peoples, irrespective of their ethnicity or outward appearance. An additional concern is that when the findings of population genomic studies do not correspond with cultural beliefs/social narratives, this may also cause group harm and/or create mistrust in the utility of genomics in such societies. The filing of patents and the potential commercialisation of research findings is an additional issue for genomic research in developing countries [53], and raises the concepts of benefit sharing and genomic sovereignty [65]. Researchers need to be prepared for this possibility when conceptualising their research.

#### Availability of resources in Africa

Conducting genomic research in resource-limited settings, such as those in Africa, offers a unique set of challenges. Comprehension barriers to the informed consent process may be present due to the limited income and literacy levels of participants as well as language differences that may be encountered [54]. For example, this is illustrated by the fact that 30.5% of the world’s living languages have an African origin, yet these diverse languages are spoken by only 12.2% of global population [66]. These concerns are further amplified in WGES studies due to the complex nature of such technologies employed [4], and the required role of IRBs/RECs: unfortunately, many board members may not be adequately trained to handle genomics-related research [36,37]. With increasing WGES in Africa over the next five years, the already limited number of local IRBs/RECs will be placed under an increased burden, with limited training and funding for these committees on the continent [67-69]. Due to the collaborative nature of WGES, ethical clearance will frequently be required from multiple IRBs/RECs, further complicating matters.

IRBs/RECs also need to determine protocols for WGES-related secondary/incidental findings in the African context. Local circumstances need to be considered, such as the feasibility of re-contacting participants (for return of results or re-consent) in certain studies as well as in resource-limited settings. Even if it may not be financially viable to undertake a time-consuming search for secondary/incidental variants [42], researchers may have an ethical obligation to return such variants if encountered during the primary research process [34]. This situation, however, is also set to change as the process of genome analysis to identify relevant variants becomes increasingly refined and automated. Another aspect that needs to be considered is that we do not currently know the penetrance range of different variants that are classified as disease-causing, which is especially relevant for African situations where understudied and unique genetic and environmental backgrounds are present [45].

Equally important is the verification step that is required before the disclosure of secondary/incidental findings to research participants. Current recommendations in the US require verification through laboratories conforming to the Federal Government’s Clinical Laboratory Improvement Amendments (CLIA); and researchers working in Africa will have to determine whether local recommendations can ensure accurate validation of findings. For example, in South Africa, the South African National Accreditation System [70] is responsible for International Organization for Standardization (ISO) accreditation of clinical laboratories in the country (e.g. ISO 15189 and ISO 17025). Where resources are limited, it may prove necessary to perform validation in foreign laboratories that comply with CLIA or equivalent protocols.

Once the relevant variants have been confirmed via verification, qualified genetic counsellors should return these results at an appropriate level for full participant understanding. The limited numbers of genetic counsellors in Africa may hamper this process. A recent review noted that only 26 genetic counsellors were listed on the relevant mailing list for the Southern African Society for Human Genetics [71,72], and even in developed countries the number of genetic counsellors is limited (e.g. in the US there is approximately one counsellor to 135 000 individuals) [44].

In the context of resource poor countries (like many in sub-Saharan Africa), the issue of what is ‘actionable’ becomes much more complex than in Western nations. Assuming availability of WGES findings, there is limited capacity to provide genetic counselling, trace and contact family members, institute preventive measures (when applicable) and follow up patients and their families. Therefore, the lack of resources and the generally poor health care systems may limit (and thereby define) what is actionable in WGES findings. Autonomy may also be hampered in such situations, where it would be difficult to convey complex concepts, such as actionable findings, to research subjects when genomic literacy is limited. There is a lack of empirical evidence on the attitudes towards the return of secondary/incidental in indigenous African populations, which should be addressed in future studies. Emerging evidence from studies of African American attitudes towards WGES suggest that these individuals are less likely to want to receive genetic results [73].

Limitations to addressing such ethical considerations for WGES in Africa thus include poor literacy and communication resources for the informed consent process; poor financial resources for secondary analysis, limited resources for appropriate validation and return of secondary data to participants, and limited capacity for health interventions based on secondary findings. Investing in Africa by providing funding for ethics processes within WGES research, however, will aid in capacity building on the continent and address some of these limitations.

#### Implications for informed consent and data sharing processes in Africa

Diverse populations, such as those found in Africa, require unique considerations for the informed consent process to acquire valid consent [50]. We wished to understand how informed consent with African participants was undertaken for previously published African genome studies, and requested access to templates of informed consent documentation from a number of different genomic studies involving indigenous African populations, in order to analyse key concepts that were covered in these documents. In general, the informed consent form templates used for the genomic studies in Africa are not available to external researchers, except for those used by large-scale projects of human genetic variation (i.e. the HapMap Project [31] and the 1000 Genomes Project [74]) as well as the Malaria Genomic Epidemiology Network (MalariaGEN) [75]. Attempts at accessing informed consent documentation from the corresponding authors of studies where these templates were not publically available proved generally unsuccessful. This may have been due to institutional policies (e.g. template consent form documentation could perhaps be considered proprietary information).

Informed consent processes for genomic research in African populations have previously been brought under scrutiny [76], highlighting the need to carefully consider these processes and ensure that the forms cover the required concepts and are written in an accessible language. Furthermore, the processes and documentation for informed consent should be available to all interested parties, in the interests of transparency. The potential linguistic barriers in Africa were mentioned previously [77], but these may be more easily overcome if scientific jargon is avoided while using language familiar to the participants [53]. Local vernacular terms that have potential derogatory connotations to participants should be avoided, distinctions between clinical care and research should be articulated and it should be emphasised that research participation is voluntary [78]. Where possible, local field workers should understand the study adequately in order to perform recruitment and should engage in feedback to other members of the research team [79].

Efficient methods to convey complicated concepts during the informed process are required to minimize inconvenience for research participants. Persons entrusted to gather informed consent should verbally go through the documentation in the home language of the research participants, who should also be given sufficient time to consider whether they wish to participate in the study. Audio- or video-recorded verbal consent could be used in certain situations where research participants are illiterate [80].

With regards to data sharing, policies that were established for the MalariaGEN, which conducted genomic research in numerous sub-Saharan African countries, may serve as a primer for future WGES studies in Africa, especially those involving large consortia [81]. Acceptable uses of data and data sharing for different parts of MalariGEN are determined by an independent data access committee, in consultation with local IRBs/RECs, while considering the respective consent that was obtained [82]. Finally, the network has adopted a noteworthy approach to intellectual property issues, where potential royalties will go to participating communities and not the investigators where possible [75]. All ethics documentation, including the informed consent templates, are available to the public.

In the subsequent section relevant South African legislation for WGES studies will be discussed in relation to UK and US laws, using this case study to gain a better understanding of potential legal issues in African countries. South Africa has been chosen not only for its relative convenience (being known by the authors), but also due to the fact that it often serves as a legislative blueprint for other (especially Anglophone) African countries, and is therefore a pertinent point of departure for further research and comparative analysis within the greater African continent.

### Legal issues around privacy, informed consent and data sharing

From a legal point of view, the two most important subject rights with regard to medical research are the right to bodily integrity and the right to privacy. The former right pertains to the methods used in acquiring samples for research, and legislation such as the National Health Act 61 of 2003 of the Republic of South Africa regulates the position. In this regard, written informed consent is required for the removal of biological material for use in genetic research. The latter right relates to how any data derived from such research should be dealt with, which is the focus of this paper.

The right to privacy has been most famously described as the right to be left alone [83], and includes the right to control the dissemination of information about one’s private life. In the modern context, the right to privacy is guaranteed either expressly, in the Constitution of the Republic of South Africa, 1996, or the UK’s Human Rights Act, 1998, for example, or implicitly - as is the case in the USA, where the Supreme Court stated in Griswold v Connecticut 381 U.S. 479 (1965) that the right is inferred. In addition, the right to privacy can also be protected through private civil action, either by means of delict/tort, a breach of confidence, or contractual remedies (see National Media v Jooste 1996 (3) SA 262 (A); R v Department of Health [2001] Q.B. 424; Restatement (Second) of Torts, American Law Institute, 1977).

To give effect to privacy rights, many countries have adopted legislation not only to provide a general framework, but also specifically to deal with the instances relating to medical research. Accordingly, we shall discuss and evaluate these legislative measures, looking at South Africa, the UK (as both a sovereign state as well as a member of the EU), and the USA. This is done in order to ascertain to what extent South Africa adheres to international standards, and to identify what steps, if any, can be taken in order to bring it in line with them.

With regard to specific legislation dealing with medical ethical concerns, the South African position is regulated by the National Health Act 61 of 2003, which primarily concerns itself with aspects relating to the right to bodily integrity. Furthermore, Regulation 13 of the Regulations relating to the use of Human Biological Material (2012) provides some guiding principles in relation to the storage and flow of genetic information. In this regard, institutions that keep or disclose genetic material records and other individually identifiable or related health information must ensure, among other things, that the information is treated confidentially and anonymously if used for research purposes, and that written informed consent is obtained before a person’s specific information is released to any relevant person. In the USA, the Health Insurance Portability and Accountability Act (HIPAA) of 1996 provides regulations relating to privacy and security of patient-client records, but does not cover de-identified information. Additionally, the Patient Safety and Quality Improvement Act of 2005 contains more stringent regulations regarding patient information to increase patient safety. It would seem, therefore, that equivalent protection is granted in the USA, although seemingly only insofar as it relates to the doctor-patient relationship, rather than the position of genetic research participants, and, while possible, it is unsure if the legislation would be interpreted extensively enough to include them as well. Some of these concerns were also recently raised in a US report on privacy and progress in whole genome sequencing [84]. In the UK, no additional specific legislation apparently exists regarding privacy in genetic research, although case law on this issue indicates that the primary concern of the law is to protect the confider/patient’s personal privacy, which can be safeguarded through anonymisation (see R v Department of Health [2001] Q.B. 424; Common Services Agency v Scottish Information Commissioner [2008] UKHL 47). It would seem, therefore, that, in general, if anonymity can be guaranteed, the use and transfer of databases containing WGES research should not be overly problematic. However, in instances where individual identification (or rather, re-identification) is possible, additional measures would have to be implemented.

Over and above the privacy concern, an additional risk that can potentially manifest would be that the information obtained could be used to discriminate against a research participant. In this regard, specific protection is directly guaranteed in the USA through the Genetic Information Nondiscrimination Act (GINA) of 2008, and indirectly in South Africa through the Employment Equity Act 55 of 1998 and the Promotion of Equality and Prevention of Unfair Discrimination Act 4 of 2000. The UK does not, however, provide legal protection in this regard, and, therefore, confidentiality and the protection of personal information become critical issues [85]. Accordingly, the general legislative framework surrounding this aspect will be evaluated.

It is submitted that the UK’s lack of directly pertinent legislation (as shown above) should not be a concern, due to the broad protection granted through the Data Protection Act, 1998 (which is the localised implementation of EC Directive 95/46, the so-called Data Protection Directive). The Act seeks to protect the privacy of any information relating to a living individual who can be identified from the data itself or from the data and other information, which is in the possession of the controller of such data. In terms of Schedule 1 to the Act, certain data protection principles (which are universally applicable in the EU) must be adhered to, including that data must be processed fairly and lawfully. Accordingly, data should be processed subject to the purposes for and the conditions under which it was acquired, while adhering to all relevant legal principles (be they found in legislation, conventions, or otherwise), and also bearing in mind the nature of the data itself. With regard to the latter aspect, a distinction is drawn between merely personal data and sensitive data. Sensitive data, in terms of Schedule 3, includes information relating to the race or ethnic origin of a person, or their physical or mental health. In terms of the Act, fair and lawful processing of such data entails that the data subject give explicit consent unless, among other things, the information is necessary to protect the vital interests of data subject or another person, deliberately made public by the data subject, or is used by a health professional and is necessary for medical purposes. In this regard, it is submitted that WGES research could be seen to be sensitive data that would require explicit consent. This notwithstanding, it must be noted (as shown above) that UK courts are of the opinion that sufficiently anonymised data would not be problematic.

In general, data protection in South Africa is currently regulated by Chapter VIII of the Electronic Communications and Transactions Act 25 of 2002, which only deals with information obtained through electronic transactions, and which, therefore, would in all likelihood not apply to most instances of WGES research. Additionally, the specific provisions of the Act have elicited other criticism [see Van Der Merwe et al. [86]] due to the fact that the data protection principles do not adhere to acceptable international standards (for instance, no special treatment is afforded for sensitive data) and are voluntary in nature. To address these shortcomings, new legislation has been proposed in the form of the Protection of Personal Information Bill (B9-2009), which provides for greater security, openness and accountability, and also creates a distinction between standard and ‘special personal’ information which would include information relating to WGES research, and would require explicit consent for purposes of processing. Accordingly, the Bill, if enacted, will bring South African data protection laws in line with EU standards, and also serve to strengthen the current framework for privacy in WGES research in South Africa.

In contrast to the stringent regulation found in the European Union, and the subsequent South African attempts to keep in step with these requirements, the USA has no specific protection regarding data privacy of individuals over and above what has already been stated, except for incidental regulation [87]. The fact that there is no international harmony in this regard is problematic, as both the UK Data Protection Act and the EU Data Protection Directive provide that no transfers of data to outside the European Economic Area may be made unless adequate protection is guaranteed. Accordingly, if the stated principles are not adhered to, this could constitute a barrier to both transnational research and access to research information.

As a compromise in the above situation, an exception exists in both the Act and Directive to allow transfers to third countries if, among others, a data subject gives unambiguous consent to a transfer – which consent must be freely given, specific and informed - or the transfer is on terms approved or authorised as ensuring adequate safeguards. In this regard the European Commission has developed prescribed standard contract terms for data transfer agreements (Commission Decision 2010/87/EU), as well as a method whereby a multinational corporation can develop binding corporate rules that, subject to approval through EU cooperation procedures, would ensure acceptability of a transfer (for more on this, see [88]). Finally, the USA and EU have also entered into a so-called ‘Safe Harbour’ agreement (Commission Decision 2000/520/EC), which encompasses a set of voluntary, self-regulatory principles in order to assist in facilitating data transfer.

It would seem that the privacy concerns relating to WGES research are not overly problematic in most countries, as long as the anonymity thereof can be guaranteed. Forgó et al. [89] point out that this is, however, not so simple, because it is necessary to keep an identifiable link between a donor and his genetic data to be able to give feedback on relevant findings connected to the donor’s genetic data, and also when one takes into account the uniqueness of genetic data. As a result, true anonymisation is not a viable option for genetic research. Lowrance and Collins [19], identify methods of mitigating this, such as through limited release of genome segments, but also indicate how this may pose its own problems for research. Thus, it would seem that the most prudent route is still to obtain express written consent from research participants. This process does not come without its problems, as express consent is required to be freely given, fully informed, and, with reference to processing and transfer of data, must be specific in nature. This means that the use of informed consent forms that are overly broad or vague may end up creating instances where researchers believe themselves to be operating lawfully, when technically they are not. This is further exacerbated, both legally and ethically, in the African context, where often research is conducted using participants who may not be literate or fully comprehend the nature and extent of the consent which they purport to give.

### Recommendations for future genomic studies in Africa

Future WGES studies in African populations need to consider the points highlighted above in order to perform appropriate research. Although not all-inclusive, this would be aided by the development of local WGES-related policies (including the promotion of sharing informed consent documentation), as well as the investment in relevant training and the development of educational aids. A variety of useful online resources for ethical and legal considerations for WGES studies in African populations can be found in Table 1.

**Table 1:** Useful online ELSI resources, especially relevant for performing research in African populations

**Resource**	**Link**
H3Africa High-Level Principles on Ethics, Governance and Resource Sharing	http://h3africa.org/ethics_governance_resourcesharing.cfm
Informed Consent: Ethical Considerations for Investigators Proposing to Collect Samples for the H3Africa Program	http://h3africa.org/informedConsent.cfm
NIH National Human Genome Research Institute: Informed Consent for Genomics Research	http://www.genome.gov/27026588
NIH Office of Extramural Research: Protecting Human Research Participants Course	http://phrp.nihtraining.com/users/login.php
About the 1000 Genomes Project (Informed Consent Template)	http://www.1000genomes.org/about
Training and Resources in Research Ethics Evaluation	http://elearning.trree.org
MalariaGEN: Ethics and Governance	http://www.malariagen.net/community/ethics-governance
Southern African Society for Human Genetics: Documents and Links	http://www.sashg.org/documents.htm

#### Development of local WGES-related policies

The development of local policies and legislation that are relevant to WGES research (e.g. informed consent, data sharing, and the return of results) will be essential for performing successful genomics research in Africa. It is recommended, as far as possible, that standardised methods to obtain informed consent from research participants should be developed in order to ensure that a generally applicable and recognisable procedure is established. It is advisable not only to have these forms drafted in the lingua franca of research participants, but also in language which is clear, succinct and easy to understand. Even when not dealing with illiterate participants, it is generally best practice to explain the nature and extent of the informed consent document in order to fully ensure observance with both the letter and the substance of the laws surrounding research and data protection. In the instance where one does deal with illiterate participants, it is technically possible to obtain written consent through the use of a thumbprint or other identifying mark if one is able to provide proof that the ‘signed’ document has been adequately explained to the participant. In this regard, additional information providing means, such as video recordings, would be advised.

Given that there is a movement towards greater and more open access to WGES research and findings, it is in the best interests of all involved to share novel ways and best practices in order to develop a universally applicable standard in this regard. This is particularly relevant for templates of the informed consent documentation that have been employed in WGES studies in African populations that have successfully been through the IRBs/RECs review process. It has been suggested that that this could be achieved through dedicated public databases for such templates and/or the inclusion in supplementary material [4]. Presently, the lack of transparency in informed consent documentation at the levels of individual researchers and universities, and that many journals do not appear to currently have policies that promote the availability of informed consent templates and standard operating procedures, may hamper researchers in resource-limited settings designing robust WGES informed consent documents. Most journals that publish genomic research insist on studies that are ethics-compliant; a further, positive step, however, would be to request templates and documentation of the informed consent process to be publicly available on publication of the study. Journals should therefore encourage this practice in a similar manner that it has been supported for genomic genotype-phenotype and gene expression datasets.

The sharing of this documentation should not, however, be an attempt to create ‘one-size-fits-all’ or ‘boilerplate’ informed consent documents, since this would not be feasible for diverse African situations; but rather as another resource for researchers to aid in the design of appropriate WGES research. Relevant, rapid assessment processes in rural communities prior to the commencement of genomic studies in particular communities can used to subsequently tailor the consent process in a culturally appropriate manner [78,90]. Such surveys could aid future African WGES studies in the conceptualisation stages of genomic research.

The development of WGES data sharing and data release policies will be essential for future genomics research on the African continent and should be performed in close consultation with stakeholders. African funding agencies, such as the South African National Research Foundation and the South African Medical Research Council, do not currently require genomic data to be made publically available. Clear WGES data sharing policies that are relevant for local research should therefore be drafted by such agencies in the future, making sure to adhere to internationally accepted standards.

Data security is of paramount importance for WGES studies to prevent surreptitious use of data and it is therefore essential that such data be protected in researchers’ personal as well as public databases. High standards of data protection should therefore be commonplace for genomic studies, including those involving WGES. Further, databases of biorepositories should also have adequate infrastructure to prevent data sensitive information from being leaked as well as data intrusion. The use of material transfer agreements will help prevent samples being used for purposes not originally agreed upon and therefore aid in avoiding inappropriate research when genetic samples are transferred between research groups, especially when the genomic analyses of African samples are being performed abroad [53,64,91].

Finally, IRBs/RECs need to develop qualified results disclosure policies as well as criteria that indicate what type of secondary/incidental research findings, if any, need to be returned to research participants. The kind of relationship that exists between the researchers and the research participants could influence this [35].

#### Training and educational materials

Providing infrastructure for various levels of training in the relevant concepts of the applications of NGS technologies in human research will be important on the continent of Africa, especially since there is a need to improve genetic literacy in sub-Saharan Africa [92]. Workshops for training members of IRBs/RECs as well as interested researchers will aid in ensuring informed ethical review processes for WGES studies. Public lectures/information sessions could be used to educate the community and gauge the public perception of WGES studies. Internationally, the ethical and legal concepts raised by WGES studies in humans are gaining attention [84,93]. Recommendations from the recent October 2012 US Presidential Commission for the Study of Bioethical Issues Report, “Privacy and Progress in Whole Genome Sequencing” [84], should be analysed and used to aid in drafting recommendations for African situations. Low educational levels of research participants with regards to genetic/genomic concepts may require the development of unique educational materials to be used during the informed consent process. Interactive explanations and the use of novel technologies to explain complicated concepts to research participants will be integral in this regard. For example, the integration of emerging software applications (i.e. apps), similar to the Illumina MyGenome iPad app [94], could be used during the recruitment stage of WGES studies.

An encouraging sign for future WGES studies in Africa is the investment in an Ethical, Legal, and Societal Issues (ELSI) Research Program by the H3Africa Initiative, which will make almost $2 million in funding available, over a period of three years [95]. The Southern African Human Genome Programme, which will be involved in the sequencing of African genomes and has received provisional funding from the South African Department of Science and Technology, also aims to highlight relevant ELSI topics brought up by such research [96]. Finally, meetings such as the Ethics and Genomics Research in Africa (EAGER-AFRICA) Conference [97] will positively contribute to the field by bringing researchers from across Africa and the rest of the world together and facilitate networking and collaboration.

### Ethical, legal and societal issues (ELSI)-related research needs in Africa

While a number of ELSI-related research studies have now been conducted in Africa [for example, see Marshall et al. [77], MalariaGEN [81], Tekola et al. [62,78], Nyika [69], Marsh et al. [79]], more studies are urgently needed. Such ELSI research could include:

Issues of informed consent, data sharing, identifiability and disclosure of secondary/incidental findings in WGES studies in contrast to GWAS or candidate gene studies in Africa.

Studies of how genomics findings are perceived in relation to ethnicity and identity in sub-Saharan Africa.

Ethical and legal aspects of biorepositories and long-term storage/distribution of biological material for genomic studies.

Analysis of how IRBs/RECs in African countries and institutions currently review WGES studies.

The relevance of local African legislation and governmental policies in the context of WGES studies in African populations.

These and some other issues are highlighted in the 2012/13 H3Africa call for applications for ELSI research programs (mentioned in the previous section). A summary of various ELSI issues faced by WGES in Africa raised in this study as well as potential recommendations to help address some of the aspects can be found in Table 2.

**Table 2:** Challenges and recommendations for ethical and legal issues for performing WGES studies in Africa

**Issue**	**Challenge**	**Recommendation**
Informed consent and data sharing	Limited availability of informed consent documentation templates for genomic studies in African populations	Journals and researchers should promote the public availability of these documents
	Cultural and ethnic diversity in Africa	Community engagement, rapid assessment processes and local field workers
	Language differences	Ensure the use of translators who are aware of cultural sensitivities
	Limited data on local views on genomic data sharing and related concepts (e.g. privacy and stigmatisation)	Generate empirical data on these concepts and opinions through relevant research
Disseminating secondary findings	Lack of local policies and guidelines	Perform studies on African research participants’ attitudes towards return of WGES results. Encourage IRBs/RECS to develop such documentation
	Insufficient genetic counsellors and bioinformaticists	Training programmes and the creation of posts for these professions
	Limited understanding of the penetrance of genetic variants in African populations with unique genetic backgrounds and environmental exposures	Investment in African WGES medical research and related infrastructure
Development of local legislation	Current legislation is not drawn up with medical WGES research in mind	The development of local legislation that is relevant for WGES research and in line with international best practices
	Numerous African countries with different legislation	Conduct jurisprudence studies that analyse relevant legislation in understudied African countries
Limited resources	Poor education/literacy levels	Development of unique, accessible educational materials
	Limited numbers of IRBs/RECs and many members are not familiar with genomic principles	Perform training workshops and ensure that funding is allocated to these groups
	Ability to perform the equivalent of CLIA-validation in local laboratories may be lacking	Analyse local laboratory standards and perform validation in foreign laboratories if necessary

## Summary

It is essential to perform WGES studies in African populations to ensure that the benefits of genomic medicine are available to all global populations. This is especially important since NGS technologies may avoid the potential pitfalls of DNA microarray-based GWAS in these individuals (e.g. low marker and linkage disequilibrium coverage) [98]. Sustainable WGES research requires strong multi-disciplinary collaborations, and in Africa will require the principle of reciprocity to be embraced by researchers and the avoidance of the one-sided South to North shipment of samples that has been frequently encountered in the past. One of the benefits of the H3Africa Initiative is that collaborations will be strengthened not only between Africa and the rest of the world, but between African countries themselves. Researchers should act as stewards for research participants and transparent research should be promoted to ensure researcher-participant trust.

Key WGES study stakeholders, such as researchers, participants and the public, need to be consulted if society is going to readily adopt precision medicine in the future. Empirical research and conceptual analyses in African populations regarding identifiability risks for participants in WGES studies need to be performed as well as participants’ preferences with respects to these concepts are required. Currently, research participants should give express consent for data sharing and in the future, studies of attitudes towards the concept of privacy in relation to WGES research in Africa need to be conducted. Creating a balance between the risks (e.g. social stigmatisation and individual privacy) and the benefits (e.g. advancement of research and clinical applications) of WGES research will ensure a sustainable future for these studies. To conclude, local ethical and legal frameworks that are appropriate for the WGES era of research are urgently required in Africa. However, until these guidelines are in place, IRBs/RECs will have to analyse the eligibility of different studies on a case-by-case basis.

### Case law references

United Kingdom:

Common Services Agency v Scottish Information Commissioner [2008] UKHL 47

R v Department of Health [2001] Q.B. 424

United States of America:

Griswold v Connecticut 381 U.S. 479 (1965)

Republic of South Africa:

National Media v Jooste 1996 (3) SA 262 (A)

### Legislation and Legislative Instruments

European Union:

Directive 95/46/EC on the protection of individuals with regard to the processing of personal data and on the free movement of such data

Commission Decision 2010/87/EU on standard contractual clauses for the transfer of personal data to processors established in third countries under Directive 95/46/EC of the European Parliament and of the Council

Commission Decision 2000/520/EC pursuant to Directive 95/46/EC of the European Parliament and of the Council on the adequacy of the protection provided by the safe harbour privacy principles and related frequently asked questions issued by the US Department of Commerce

United Kingdom:

Data Protection Act, 1998

Human Rights Act, 1998

United States of America:

Genetic Information Nondiscrimination Act of 2008

Health Insurance Portability and Accountability Act of 1996

Patient Safety and Quality Improvement Act of 2005

Restatement (Second) of Torts, American Law Institute, 1977

*Republic of South Africa*:

Constitution of the Republic of South Africa, 1996

Electronic Communications and Transactions Act 25 of 2002

Employment Equity Act 55 of 1998

National Health Act 61 of 2003

Promotion of Equality and Prevention of Unfair Discrimination Act 4 of 2000

Protection of Personal Information Bill, B9-2009

Regulations relating to the use of Human Biological Material, 2012

## Abbreviations

CLIA: Clinical Laboratory Improvement Amendments; dbGaP: Database of Genotypes and Phenotypes; EAGER-AFRICA: Ethics and Genomics Research in Africa; EGA: European Genome-phenome Archive; ELSI: Ethical, Legal, and Societal Issues; EU: European Union; GINA: Genetic Information Nondiscrimination Act; GWAS: genome wide association studies; H3Africa: Human Heredity and Health in Africa; HIPAA: Health Insurance Portability and Accountability Act; IRB: institutional review board; ISO: International Organization for Standardization; MalariaGEN: Malaria Genomic Epidemiology Network; NGS: Next generation sequencing; REC: Research ethics committees; WES: Whole exome sequencing; WGES: Whole genome and whole exome sequencing; WGS: Whole genome sequencing.

## Competing interests

The authors declare that they have no competing interest.

## Authors’ contributions

GEBW wrote the first draft of the manuscript excluding the legal section, which PGJK wrote. AAA and NT contributed towards analysis of the literature and critically revising the manuscript. All authors read and approved the final manuscript.

## Pre-publication history

The pre-publication history for this paper can be accessed here:

http://www.biomedcentral.com/1472-6939/14/21/prepub
